# Altered right anterior insular connectivity and loss of associated functions in adolescent chronic fatigue syndrome

**DOI:** 10.1371/journal.pone.0184325

**Published:** 2017-09-07

**Authors:** Laura Anne Wortinger, Merete Glenne Øie, Tor Endestad, Vegard Bruun Wyller

**Affiliations:** 1 Department of Paediatrics and Adolescent Health, Akershus University Hospital, Nordbyhagen, Norway; 2 Department of Psychology, University of Oslo, Oslo, Norway; 3 Research Department, Innlandet Hospital Trust, Lillehammer, Norway; University of Texas at Austin, UNITED STATES

## Abstract

Impairments in cognition, pain intolerance, and physical inactivity characterize adolescent chronic fatigue syndrome (CFS), yet little is known about its neurobiology. The right dorsal anterior insular (dAI) connectivity of the salience network provides a motivational context to stimuli. In this study, we examined regional functional connectivity (FC) patterns of the right dAI in adolescent CFS patients and healthy participants. Eighteen adolescent patients with CFS and 18 aged-matched healthy adolescent control participants underwent resting-state functional magnetic resonance imaging. The right dAI region of interest was examined in a seed-to-voxel resting-state FC analysis using SPM and CONN toolbox. Relative to healthy adolescents, CFS patients demonstrated reduced FC of the right dAI to the right posterior parietal cortex (PPC) node of the central executive network. The decreased FC of the right dAI–PPC might indicate impaired cognitive control development in adolescent CFS. Immature FC of the right dAI–PPC in patients also lacked associations with three known functional domains: cognition, pain and physical activity, which were observed in the healthy group. These results suggest a distinct biological signature of adolescent CFS and might represent a fundamental role of the dAI in motivated behavior.

## Introduction

Estimates regarding the prevalence of Chronic Fatigue Syndrome (CFS) during adolescence ranges between, 1% and 2%, depending on methodology and diagnostic criteria [1–4]. The central ailments of CFS are abiding and debilitating fatigue accompanied by cognitive impairments, physical and mental activity intolerance, and pain [5]. Autonomic nervous system dysfunction [6], alterations in facilitatory and inhibitory pathways [7, 8], and abnormalities of the neuroendocrine system [9–11] in CFS support the notion of a disorder in which an interplay of neural and endocrine factors might attribute to aberrant neurobiological stress responses—*sustained arousal* [12]. Our research group has further suggested that adolescent CFS is associated with alterations in brain connectivity, wherein abnormalities influence fatigue awareness [13].

Regional functional connectivity patterns of the right dorsal anterior insula (dAI) are currently missing in the literature for CFS, and a better understanding of its connectivity could shed light on the integrity of neurocognitive network dynamics in adolescent CFS. The right dAI is a primary hub of the brain’s salience network (SN), which has been associated with interoceptive awareness [14–17], and control signaling for the engagement of central executive network (CEN) [18–21]. The CEN contains the dorsolateral prefrontal and lateral posterior parietal cortices and alterations in this network reflect impaired cognition (i.e. working memory and executive control functions) [22–26].

The right dAI functional connectivity has been implemented in disorders where there appears to be a disruption in the interpretation of important bodily information: chronic pain [27–31], irritable bowl syndrome [32, 33] and depression [34, 35]. Decreases in the right dAI–posterior parietal cortex (PPC) functional and structural connectivity have been associated with impoverished cognition in younger children; furthermore, maturation of the functional coupling between these key SN and CEN nodes is suggested to underlie cognitive control development [20]. Additionally, the right dAI might serve as an important biomarker that provides important information about treatment specificity and success [35].

High-level attention and cognitive control processing require efficient interactions of the brain’s SN and CEN. In CFS, network investigations have robustly identified alterations in functional connectivity (FC) of SN in both adolescent and adult studies [13, 36–38]. Particularly, FC decreases to the right insula [13, 37, 38], which associate with fatigue severity [13]. FC decreases in the CEN have been reported in adult CFS studies [36, 37], but an adolescent CFS study did not find CEN alterations [13].

In a previous independent components analysis, we found SN FC decreases to the right insula [13], but results did not include the right dAI, which corresponds most closely with the AI hub of the SN [39]. Network hubs are vulnerable to pathology and considered biologically costly [40]. The normal hierarchical architecture of brain networks is disrupted as a result of hub deficiencies in several neurological diseases [41]. Previous MRI reports in CFS have suggested that regulatory brain regions themselves might be unaffected, but a collective dysregulation has been observed in two-way signaling and correlated functions [42, 43]. We have reported that cognition [44], pain [45] and physical activity [46] are three domains of impaired function in adolescent CFS. These functional domains are likewise associated with the efficiency of right dAI connectivity and cognitive control in healthy participants [15, 17, 20, 47–49].

In a second analysis on this common data set [13], the aim of the current study was to investigate the regional connectivity of the right dAI in adolescent CFS patients compared to a healthy comparison group. Secondly, we explored the relationship between right dAI functional connectivity and three domains of function: cognition, pain, and physical activity.

## Method

This study is part of the NorCAPITAL-project (The Norwegian Study of Chronic Fatigue Syndrome in Adolescents: Pathophysiology and Intervention Trial) (Clinical Trials ID: NCT01040429). It was conducted at the Department of Pediatrics, Oslo University Hospital, Norway, which is a national referral center for young CFS patients. The current study is based on cross-sectional data collected during the first clinical in-hospital day of NorCAPITAL, from March 2010 to May 2012. All participants received a gift-card worth NOK 200. Informed, written consent was obtained from all participants and from parents/next-of-kin if required. The study was conducted in accordance with the Helsinki Declaration and approved by the Norwegian National Committee for Ethics in Medical Research.

### Participants

All hospital pediatric departments in Norway (n = 20), as well as primary care pediatricians and general practitioners, were invited to refer CFS patients aged 12–18 years consecutively to our department.

The referring units were equipped with written information for distribution to potential study participants and their parents/next-of-kin. If consent was given, a standard form required the referral unit to confirm the result of clinical investigations considered compulsory to diagnose pediatric CFS (pediatric specialist assessment, comprehensive hematology and biochemistry analyses, chest x-ray, abdominal ultrasound, and brain magnetic resonance imaging) [50]. Also, the referring units were required to confirm that the patient a) was unable to follow normal school routines due to fatigue; b) was not permanently bedridden; c) did not have any concurrent medical or psychiatric disorder that might explain the fatigue; d) did not experience any concurrent demanding life event (such as parents’ divorce) that might explain the fatigue; e) did not use pharmaceuticals (including hormone contraceptives) regularly. If medical history or current health status indicated a psychiatric condition, physicians were required to refer patients to a psychiatrist for evaluation. If a comorbid psychiatric disorder was found, those patients were removed from the study [46]. No patients received graded exercise therapy and two patients (out of the 18 viable resting-state MRI datasets) received cognitive behavioral therapy at baseline. Completed forms were consecutively conveyed to the study center and carefully evaluated. Patients, considered eligible for this study, were summoned to a clinical meeting at our study center, and after which, a final inclusion decision was made.

In agreement with NICE clinical guidelines [50, 51], we applied a ‘broad’ case definition of CFS, requiring three months of unexplained, disabling chronic/relapsing fatigue of new onset. We did not require that patients meet any other accompanying symptom criteria, in contrast to the case definition from the International Chronic Fatigue Syndrome Study Group at the Centers for Disease Control and Prevention (commonly referred to as the Fukuda-definition), which appears to be most frequently used in the scientific community [52]. The Fukuda-definition requires at least six months of unexplained chronic or relapsing fatigue of new onset, severely affecting daily activities, as well as four or more of eight specific accompanying symptoms (headache, muscle pain, joint pain, sore throat, tender lymph nodes, impaired memory or concentration, unrefreshing sleep, and malaise after exertion). However, the validity of this definition has not been established [53]. In fact, several empirical findings raise concerns about the validity, in particular among adolescents: A formal factor analysis of symptoms in a broadly defined group of chronic fatigued patients did not show a strong correspondence with the Fukuda accompanying symptoms [54]. A study based upon the Swedish twin registry concluded that there was no empirical support for the requirement of four out of eight Fukuda accompanying symptoms [55]. A report on a broadly defined population of adolescent CFS patients concluded that the subgroup adhering to the Fukuda criteria was not characterized by a certain level of disability, nor was this subgroup specifically related to characteristics of underlying pathophysiology (alteration of cardiovascular autonomic control) [56]. Accordingly, subgrouping based upon the Fukuda criteria did not influence the cross-sectional comparisons or the intervention effects in previously reported results from the NorCAPITAL project [46]. Thus, the inclusion criteria in this study are wider than the Fukuda criteria. The main reason for not adhering to the Fukuda case definition was too few accompanying symptoms.

In NorCAPITAL, a total of 120 CFS patients were included. This study is based upon a subset of patients generated from a computer-based randomization procedure, where one fourth of the patients were randomized to be included in the present study; 18 months disease duration served as stratification criterion [46]. The randomization procedure allocated 30 patients to fMRI assessment: of these, five patients did not want to participate in the present study, four patients were excluded due to orthodontic treatment, two participants were removed due to scanning error, and one was excluded due to excessive movement > 3 mm in either of the three translation parameters or three rotation parameters, resulting in a total fMRI dataset of n = 18 adolescent CFS patients (mean age 15.9 years) for the final analyses. A group of 18 healthy controls (mean age 15.9 years) having a comparable distribution of gender and age were recruited from local schools. No chronic disease and no regular use of pharmaceuticals were allowed. Symptom data were missing at random for two of the patients, and the group mean was used for their lost data.

### Clinical measures

#### Fatigue

The Chalder Fatigue Questionnaire is a valid outcome measure in both adult [57] and adolescent CFS [58]. It is based on symptoms during the preceding month. The sum across 11 items is scored on a 0–3 Likert scale, thus ranging from 0 (less severe fatigue) to 33 (more severe fatigue).

#### Depression

The Mood and Feelings Questionnaire (MFQ) has been validated in children and adolescents [59]. The MFQ consists of 34 items to be self-rated by the children or adolescents based on symptoms during the preceding month. Each item is scored on a 0–2 Likert scale, and the total sum score is from 0 to 68. Higher scores imply more depressive symptoms.

#### Working memory

Working memory was measured by adding raw scores on the digit span forward and backward tests from Wechsler’s Intelligence Scale for Children–IV (WISC-IV) [60]. During examination, the examiner read aloud strings of random digits (approximately one digit per second). The first two strings consisted of 2 digits, the next two strings of 3 digits, etc. The digit span forward test required the test person to repeat the digits in the same order as the examiner presented; in digit span backward, the test person repeated the digits in the reverse order. Each answer is scored 1 (correct) or 0 (incorrect). When both strings in a pair (i.e. two strings of equal length) are answered incorrectly, the test is discontinued.

#### Pressure pain threshold (PPT)

The PPT is a reliable variable to test for hyperalgaesia in superficial structures such as skin, nails and underlying muscles [61]. Pressure provoked pain thresholds were mapped using a commercially available force transducer with a rubber tip of 0.5 cm2 (Algometer, JTECH, medical, Salt Lake City, Utah, USA). The fingernail of the third finger, skin superficial to the trapezius (ascending part), and supraspinatus muscles bilaterally were the three predefined sites tested, see Winger [45] for description of PPT procedure. Reduced thresholds on symptomatic as well as asymptomatic/remote places may indicate a general sensitization [8]. Averaged PPTs were summed to give a total PPT score across regions.

#### Daily physical activity (Steps/Day)

We used the activPAL accelerometer device (PAL Technologies Ltd, Glasgow, Scotland) for monitoring of daily physical activity during seven consecutive days. ActivPAL provides reliable and valid data on step number and cadence as well as time spent on walking, standing and sitting/lying during everyday activities [62, 63]. The device has also been validated in an adolescent population [64], and it is sensitive for changes of step number with time [65].

### Resting-state fMRI data acquisition

Imaging data were collected on a 3T, Phillips Achieva whole-body scanner, with an 8 channel Philips SENSE head coil (Philips Medical Systems). Functional images were obtained with a single-shot T2*—weighted echo planar imaging sequence. Imaging sequence consisting of 250 volumes with: repetition time (TR): 2000 ms; echo time (TE): 30 ms; 3mm isotropic voxels; field of view (FOV): 240 x 240 reconstructed into 80 x 80; flip angle 80°; 38 transverse slices with 0 gap and scanned in a default interleaved sequence. The slices where collected starting from the bottom of the brain, collecting all the odd number slices first (1, 3, 5…) and then collecting all the even number slices (2, 4, 6…). The total scan time was 8 minutes. Participants were instructed to close their eyes and to rest comfortably, without moving or falling asleep, during the functional scan. For the 3D scan, an anatomical image with: TR: 10462 ms; TE: 54 ms; 2mm isotropic voxels; FOV: 224 x 224; flip angle 90°: 60 transverse slices with 0 gap and scanned in the default interleaved sequence.

### Resting-state fMRI preprocessing

Images were preprocessed using CONN-fMRI Functional Connectivity toolbox (ver.15; www.nitrc.org/projects/conn) with SPM8 (www.fil.ion.ucl.ac.uk/spm/) and the default pipeline (defaultMNI), which included functional realignment and unwarp, slice-timing correction, structural segmentation and normalization, functional normalization, ART-based functional outlier detection and scrubbing, and functional smoothing (8-mm Gaussian kernel) carried out in MNI-space [66]. In-scanner motion parameters were calculated using frame displacement (FD) [67]. FD averages rotational and translational parameter differences, using weighted scaling, and was compared between groups using two-tailed independent samples *t*-test. Between group motion difference was considered significant at *P* < 0.05.

### Seed-based connectivity analysis

We calculated the spatial mean time series for the right dAI seed region of interest (ROI) in a seed-to-voxel resting-state functional connectivity (FC) analysis. FC of right dAI was determined by bivariate correlation using the CONN-fMRI Functional Connectivity toolbox (ver.15; www.nitrc.org/projects/conn). The right dAI seed was defined by previous work [18–20] with an 8 mm radius sphere centered around MNI coordinates (*x* = 39, *y* = 23, *z* = -4) using the WFU PickAtlas [68]. Between-group effects were considered significant with a cluster-level false discovery rate (FDR) correction and a correction for multiple tests on this dataset [13], *P* values less than 0.0125.

Motion poses a significant source of noise in FC analyses. None of the participants included in the present study had motion exceeding 3 mm in any direction. We addressed residual motion-related artifacts in four steps. First, functional image volumes were realigned to the mean image. Second, six motion parameters representing each of the three cardinal directions (X, Y, and Z) and rotational movement around three axes (pitch, yaw and roll) was removed with covariate regression analysis. Third, motion scrubbing was preformed using ArtRepair software (http://cibsr.stanford.edu/tools/human-brain-project/artrepair-software.html). Through this process we identified two CFS patients and three comparison participants that required censorship and additional motion artifacts were removed with covariate regression analysis. Finally, an anatomical component correction was applied using an a *CompCor* strategy for control of physiological and movement confounds [69, 70]. This denoising step applies linear regression and band-pass filtering [0.008–0.09 Hz] in order to remove unwanted motion, physiological and other artifactual effects from the BOLD signal before computing connectivity measures.

Individual participant beta values representing Fisher’s *r*-to-z transformed correlation coefficient values, where *r* is the correlation coefficient between the seed area and voxel cluster, were extracted for significant clusters using REX toolbox.

Demographic data, clinical measures, and individual FC values (seed-to-cluster z-scores) were evaluated using SPSS, version 22, (IBM Inc.; Chicago, IL). Between-group differences were considered significant at *P* < 0.05.

FC values were subjected to regression analyses to further evaluate its relationship with clinical measures. Neural FC is associated with development, specifically during adolescence [20, 71], so age was added to regression models to control for its influence on linear relationships. Since comorbid depression seems to have a greater prevalence during adolescence in CFS [72] and aberrant FC in the SN has been identified in depressed adolescents [73], depressive symptoms were also controlled for in regression analyses.

## Results

### Demographic and clinical measures

Adolescent CFS patient and comparison groups were well matched for age, gender, body mass index (BMI) and IQ; however, patients scored higher on clinical symptom scales and had less physical activity, measured in steps/day (Table 1).

**Table 1:** Demographic and clinical measures of adolescent patients with chronic fatigue syndrome and healthy comparison participants.

Characteristic	Patients with Chronic Fatigue Syndrome (N = 18)		Healthy comparison group (N = 18)		*P*
	N	%	N	%	
Female	16	89	13	72	n. s.
^Menarche	13	81	10	77	n. s.
^a^Fukuda criteria	13	81			
^b^NICE criteria	15	94			
	Mean	SD	Mean	SD	
Disease duration in months	19.1	9.8			
Age	15.9	1.5	15.9	1.6	n. s.
^c^BMI	22.8	3.4	20.6	2.7	n. s.
IQ ^d^WASI [74]	107.9	12.1	115.9	16.9	n. s.
Fatigue ^e^CFQ	19.2	6.3	9.0	4.1	<0.001*
Depression ^f^MFQ	16.1	7.8	6.7	7.7	<0.001*
Working Memory (raw scores)	15.1	3.1	16.2	3.5	n. s.
PPT	65.4	21.2	83.9	34.7	n. s.
Physical Activity	5910	2705	10519	3686	<0.001*
Motion during scanning					
Mean frame displacement^h^	0.11	0.04	0.13	0.06	n. s.

^Menarche data was missing for 3 patients (ages 13, 16 and 17) and 1 healthy participant (age 17). Only 2 healthy participants reported that they had not experienced menarche.

^a^Participants fulfilling the Fukuda-definition of CFS [52]

^b^Participants fulfilling the National Institute for Health and Care Excellence [51] definition of CFS

^c^Body Mass Index [BMI = weight(kg)/height^2^(m^2^)]

^d^Wechlser Abbreviated Scale of Intelligence-estimated full IQ

^e^Chalder Fatigue Question [57]

^f^Mood and Feelings Questionnaire for Depression [59]

^h^Frame displacement [67]

*Indicates group comparison is significant at *p* ≤ 0.05.

The χ^2^ test was used for sex; two-sample *t*-tests were used for continuous variables.

Not significant (n. s.)

### Functional connectivity analysis

**Fig 1:**
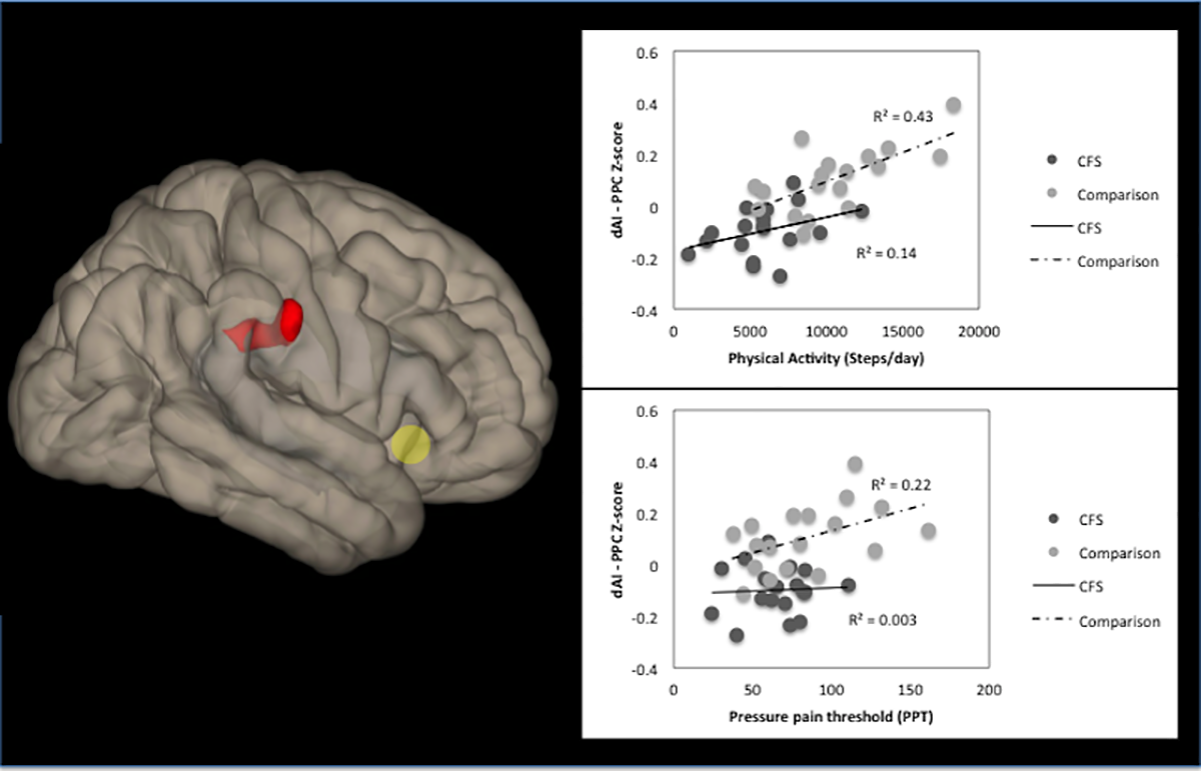
Reduced right dAI functional connectivity in adolescent CFS compared to healthy participants. Fig 1 is the right view illustrating the right dAI (yellow circle) and the location of a significant cluster (40, -32, 32), wherein connectivity was lower in the CFS group than the healthy comparison (HC) group. Regions included in the cluster were the right supramarginal gyrus, right postcentral gyrus, and right parietal operculum cortex **(Left)**. Scatter plots contain standard Z scores for FC in each group, where dark circles represent individual patients with CFS and lighter circles represent HC participants. FC between the right dAI-PPC increases with greater physical activity and pain tolerance in HC, but this relationship was not observed in adolescent CFS patients **(right)**.

Adolescent CFS patients demonstrated decreased functional connectivity (FC) with the right dAI seed in the seed-to-voxel FC analysis, relative to healthy comparison (HC) participants. Compared to CFS patients, HC subjects showed significantly greater FC of the right dAI with the right posterior parietal cortex (PPC) (Fig 1 and Table 2).

**Table 2:** Reduced right dAI functional connectivity in adolescent CFS compared to healthy participants.

Seed region	Peak-voxel Cluster coordinate	Cluster size	Cluster regions	Voxels in region	% Coverage	Cluster FDR corrected p-value	HC connectivity mean (SD)	CFS connectivity mean (SD)
Right dorsal Anterior Insula	40, -32, 32	358	Right Supramarginal Gyrus	123	15	< .0002	.105 (.13)	-.098 (.09)
			Right Postcentral Gyrus	60	2			
			Right Parietal Operculum Cortex	19	4			
			Not assigned or less than 1% coverage	156				

### Relationship between connectivity and clinical measures

For clinical domain analysis, we entered group, depression, age, working memory, PPT, and physical activity in a multiple regression model. These variables explained 72% of the variance in right dAI–PPC functional connectivity. We controlled for the effects of group, age, and depression and found working memory, PPT, and physical activity were independent predictors of right dAI–PPC functional connectivity (Table 3).

**Table 3:** Linear regression model: Working memory, pain tolerance, and physical activity predict right dAI—PPC functional connectivity.

Right dAI—PPC		Clinical domains	
		Bivariate regression	Multivariate regression
*Predictors*		*ß*	*ß* (CI)
**Group**		.683***	**0.295** (.004, .171) *
**Depression**		-.481**	-0.117 (-.006, .003)
**Age**		-.157	-0.020 (-.024, .020)
**Working memory**		.050	**-0.250** (-.021, -.001) *
**PPT**		.449**	**0.237** (.000, .002) *
**Physical activity**		.733***	**0.495** (.000, .000) **
***R***^***2***^			**0.72**
***F***			12.27***

* *p* < 0.05.

**p < 0.01.

***p < 0.001.

### Relationship between connectivity and clinical measures within groups

We found working memory, PPT and physical activity significantly predicted right dAI—PPC functional connectivity and explained 74% of the variance within the HC group. There were no significant relationships between these variables within the CFS group (Table 4 and Fig 1). In the HC group only, higher PPTs were related to increased FC of the right dAI–PPC and greater amounts of physical activity were also associated with increased FC of the right dAI–PPC. These significant relationships were observed in both simple bivariate and multivariate regression analyses of the HC group. Working memory was also a predictor in the HC group multivariate regression, but it was not significant in the bivariate regression.

**Table 4:** Linear regression models: Predictors of right dAI–PPC within adolescent CFS group and healthy comparison group.

Right dAI—PPC	CFS group			Healthy comparison group	
	Bivariate regression	Multivariate regression		Bivariate regression	Multivariate regression
*Predictors*	*ß*	*ß* (CI)		*ß*	*ß* (CI)
**Depression**	-.316	-.232 (-.010, .004)		-.064	.018 (-.006, .007)
**Age**	.176	.275 (-.029, .063)		-.463	.054 (-.034, .043)
**Working memory**	-.125	-.234 (-.024, .010)		-.083	**-.462** (-.029, -.004) *
**PPT**	.052	-.160 (-.004, .003)		.470*	**.410** (.000, .003) *
**Physical activity**	.374	.392 (.000, .000)		.659**	**.808** (.000, .000) **
***R***^***2***^		.29			**.74**
***F***		.98			6.74**

* *p* < 0.05.

**p < 0.01.

## Discussion

The principal finding of this study is that the adolescent CFS group differentiated from the healthy comparison group with decreased FC between the right dAI–PPC. A secondary finding was the lack of relationship within the CFS group between right dAI–PPC FC and function across three clinical domains: cognition, pain, and physical activity.

These results expand upon prior knowledge that aberrant SN and CEN functional connectivity patterns underlie the biology of CFS. The right dAI is part of the SN neural system that attends to biologically and cognitively relevant information and engages the CEN for working memory and cognitive control processing [18, 21, 39, 75, 76]. Intrinsic SN alterations have been identified in adult CFS [36], including regional FC decreases to the right insula [37, 38]. Adult CFS studies have also reported a reduction in intrinsic connectivity of the CEN [36] and both increases and decreases in regional FC patterns of the CEN have been found [37, 38]. Even though our previous report did not find intrinsic CEN changes in adolescent CFS patients [13], the regional FC decreases between the SN node and CEN node found in this study suggest dysfunctional interactions between brain networks.

Prior work from our group demonstrated a pattern of reduced SN FC to the right insula that was related to fatigue severity in adolescent CFS patients [13]. This posterior to anterior pattern in the right insula did not include the dAI, which corresponds most closely with the AI hub of the SN [39]. We interpreted this relationship as being associated with abnormal signaling along the right posterior to anterior insular axis that led to heightened fatigue awareness in patients. The sense of the physiological condition of the body, or interoceptive awareness, is associated right AI activity [17, 34, 77]. Interoceptive awareness is understood to result from an integration of both internal and external stimuli along a pathway from the posterior to the anterior regions of the insula [15, 17]. Deviations along this insular pathway and the SN seem to be common in disorders, such as depression, post traumatic stress disorder, and pain, where there appears to be a disruption in the interpretation of salient biological and cognitive information [39].

The current study was a re-analysis of the same sample used in a prior study but focused on another aim, namely the regional connectivity of the right dAI. The right dAI FC decreases to the PPC, a major node of the CEN neural system, suggest an inefficiency in a neural mechanism that underlies top-down cognitive control in adolescent CFS patients. We found that this implied top-down cognitive control impairment also lacked associations with three clinical domains of CFS. Physical activity [47–49], cognition [20], and pain [15, 17] are three known functions associated with efficient right dAI FC and cognitive control in studies on healthy groups.

The decreases in FC between right dAI and PPC might influence motivated behavior in adolescent CFS. It is well known that physical activity in childhood influences neural circuitry supporting high-level cognitive control (see Khan and Hillman [78] for review). An integration of cost and benefit outcomes of physical effort might derive from a motivational context provided in the AI—where worse outcomes seem to have greater representation [79]—and from the up-regulation of top-down control processes in response to motivationally salient cues [80]. Decreases in motivational neural circuitry were associated with increases in mental and general fatigue and reductions in physical activity in adult CFS [81]. Previous fMRI studies with children and adolescents with CFS found changes in activities of the prefrontal and parietal regions during attentional control [82] and decreases in striatal activity involved in reward sensitivity and motivation [83].

The relationship between right dAI–PPC FC and working memory performance was not observed in the adolescent CFS group, which implies deficient cognitive control in information processing. Cognitive skills develop significantly throughout adolescence and rely on the maturation of control processes that focus attention and allocate neural resources for efficient problem solving. One such control mechanism underlying development was discovered in the maturation of FC between brain systems of the right AI node of the SN and PPC node of the CEN [20]. The association between working memory performance and right dAI–PPC FC observed in the healthy group of our study seems to be influenced by the variance of age. Selective elimination of synapses might guide the development of FC, specifically in the SN [71], but the underlying anatomy and physiology of developing FC is still unclear. Participants’ age ranged from 13 to 18 years in this study, and in the developmental studies cited [19, 20, 71], researchers inferred FC changes during adolescence by subtracting variables from adult and child groups. The adolescent brain undergoes sophisticated neural pruning [84], which increases the specificity and efficiency of cognitive processing [85–87]. The correlation between right dAI and PPC FC and working memory performance might reflect normal neurocognitive network development in the healthy participants.

Lowered PPT in the CFS patients of our study might be an indication of a shift in circuitry thresholds, and FC decreases of the dAI with the PPC could indicate a loss of cognitive control in modulating conscious pain perception. Pain theory suggests that frontal cortical drives are embedded in corticostriatal circuits, which actively control the threshold for incorporating sensory afferent inputs into cortical conscious states, across sensory modalities [28]. Shifts in the threshold mechanisms of this circuitry influence synaptic learning-based reorganization and lowers conscious perception of pain [88, 89]. The region best related to the consciousness of pain is the AI [90], and top-down cognitive control regions modulate pain awareness in the AI [91].

The loss of connectivity and implied cognitive control over associated functions related to the right dAI might be an indication of how prolonged fatigue potentially threatens normal neurocognitive network development in adolescent CFS. It could be that fatigue and subsequent physical inactivity disrupt the maturation of functional connectivity between brain systems. Supporting this claim, alterations in white matter tracts of the right arcuate fasciculus, a bundle of long and short fibers that runs laterally to connect frontal and parietal lobes [92], was found in adult CFS [93], and might underlie the FC abnormalities of the right dAI–PPC found in the adolescent CFS patients of this study.

The right dAI might serve as a much-needed biomarker, where treatment success might be measured by improved FC and associated function across three clinical domains of CFS. As such, our findings might provide a rationale for the clinical effectiveness of cognitive behavioral therapy [94–97], and graded exercise therapy [96, 98] in CFS. These treatments may target underlying neural systems related to cognitive control, pain regulation, and motivation.

Cumulative stress decreases right insular volume [99] and alters underlying dopaminergic function [100], which is important for the modulation of motivation and cognitive control interactions [100, 101], pain [102, 103], and self-awareness [104]. Inabilities to regulate stress have been observed across multiple systems of the body and collectively comprise the *sustained arousal* model of disease mechanisms in CFS [12]. Failures to regulate stress might be the cause of right dAI connectivity dysfunction, and combined physical inactivity might again add to the FC decreases—a vicious cycle that disrupts cognition and interoceptive interpretations, and maintains the disease. Although speculative, sustained arousal might explain the functional connectivity decreases and loss of associated functions across the three clinical domains studied here in adolescent CFS patients.

### Strengths and limitations

With an adolescent CFS population, it might be easier to identify real disease mechanisms as opposed to secondary phenomena associated with years of chronicity in adults. Current research suggests that childhood CFS present differently from adults [72] and a systematic comparison of neurocognitive networks might better assess the progression of neural changes, which should be explored in future research.

A small sample size might limit the generalizability of these results; so far as can be determined, there was no reason to suspect a selection bias. Even though the sample size was small, we found significant relationships with bivariate regressions.

The relationship between the FC of the right dAI–PPC and working memory performance might require more specificity and efficiency in neural processing; therefore, age-related neural variation might have a greater influence over the refined cognitive measure than the physical measures. Normal FC development and associated functions were beyond the scope of this study. Future studies should utilize adolescent participants (instead of subtracting adult and child groups) to further characterize developing FC and address influential factors, like myelination, synaptic elimination, changing levels of neurotransmitters, and decreasing glucose metabolism and cerebral blood flow.

## Conclusion

Our findings of dysfunctional connectivity of the right dorsal anterior insula and loss of functional associations with cognitive performance, pain tolerance, and physical activity might represent a fundamental aspect in the neural architecture of adolescent CFS pathophysiology.
